# Cold anesthesia in honey bee (*Apis mellifera*) research: risks and reporting gaps

**DOI:** 10.1007/s13592-026-01280-6

**Published:** 2026-06-30

**Authors:** Lauren S. Peters, Byron N. Van Nest

**Affiliations:** Department of Biological Sciences, University of Manitoba, Winnipeg, MB Canada

**Keywords:** cooling, anesthesia, chill injury, neurophysiology, learning and memory

## Abstract

Cold anesthesia, as used in honey bee research, is often considered a safe alternative to carbon dioxide. Yet evidence in the literature shows this assumption is only partly justified. Early work demonstrated effects on behaviors such as hoarding and learning, while more recent studies report impacts on neuromodulators, memory consolidation, and locomotion. Although often nonlethal, these effects can alter physiology and behavior in ways relevant to experimental outcomes. Despite calls for greater methodological transparency, reporting of cooling protocols remains inconsistent. A review of historical and recent literature shows that although cold anesthesia is nearly ubiquitous, duration, temperature, and handling details are frequently omitted in published methods, precluding replication and complicating interpretation. While some studies indicate recovery within an hour after cooling, insect physiology research highlights risks of neural or muscular injury. Taken together, these findings suggest that anesthetic handling may be an underappreciated source of variation in contemporary studies of honey bee cognition and behavior.

## Introduction

Anesthetic methods for inducing narcosis are an important component of honey bee (*Apis mellifera* L.) neuroethological research (Human et al. 2013). They are frequently used for harnessing bees or other procedures where movement is problematic (Arslan et al. 2023). Historically CO_2_ was the standard, but mid-twentieth century studies showed it alters behavior and physiology, including increased precocious pollen foraging, reduced foraging efficacy, reduced lifespan, early oviposition in queens, and disruptions of the circadian clock (Ribbands 1950; Simpson 1954; Austin 1955; Medugorac and Lindauer 1967). In 1980, cooling was proposed as a safer alternative to CO_2_ (Ebadi et al. 1980) and was soon adopted as a simple, low-cost approach (Robinson and Visscher 1984; Tutun et al. 2020). Despite its popularity, cooling remains poorly standardized across studies. Methods vary widely (ice, refrigerators, freezers; different durations and conditions), and safety is often judged only by mortality. This overlooks the possibility of subtle damage to the nervous system or other tissues that could influence physiological or behavioral outcomes (e.g., learning, locomotion, or neuromodulator expression). Moreover, protocols are inconsistently and often incompletely reported, limiting replication and interpretation.

The risks associated with cooling have been recognized for decades (e.g., Ebadi et al. 1980) and are occasionally acknowledged in recent studies. However, researchers continue to use inconsistent methods often with limited reporting. Repeated calls to address this issue (Pankiw and Page 2003; Frost et al. 2011; Tutun et al. 2020) have yet to yield a cohesive alignment in practice. Rapid progress is now being made in understanding honey bee brains and behavior (Chittka 2022; Johnson 2023). As this research accelerates, it is critical to revisit this common technique. Without a better understanding of its impacts, new findings may not fully reflect bees’ natural physiological or behavioral states, leading to complications in comparisons between studies and potential for misinterpretation of results. This review summarizes evidence for the physiological and behavioral impacts of cooling anesthesia, evaluates consistency of reporting across studies, and highlights key research gaps that remain to be addressed to develop safe, standardized protocols within honey bee research.

## Mechanisms of Chill Coma

Cooling induces chill coma through ion-level disruptions in excitable cells, which halt neural and muscular activity (Hosler et al. 2000). This cellular shutdown can have consequences beyond immobilization, including disruptions in memory consolidation and other cognitive processes (Erber 1976; see also Menzel 2012). In this section we summarize both the cellular physiology and the cognitive effects observed in honey bees.

### Cellular and physiological mechanisms

Cooling acts as an effective anesthetic in ectothermic invertebrates. Placing insects into an ice bath, onto a cold plate, or within a refrigerator or freezer can drop their internal body temperature to a point where they become immobilized—chill coma—without necessarily causing tissue damage—chill injury (MacMillan et al. 2015). Chill coma results not from tissue injury but from disrupted cell-to-cell signaling at low temperatures. Electrophysiological recordings of muscle activity at the time chill coma is induced show a sudden sustained burst of action potentials that gradually decreases in amplitude until the cell can no longer fire (Hosler et al. 2000). This pattern appears consistently across invertebrates, though the burst duration and the temperature of chill coma onset vary by species; for honey bees, chill coma occurs at 10.6 ± 1.2 °C (SEM) (Hosler et al. 2000).

Hyperkalemia, caused by a disruption in active transport during cooling, appears to suppress action potentials after the initial burst, thereby maintaining narcosis (Rodgers et al. 2007; MacMillan and Sinclair 2011; MacMillan et al. 2015; Bayley et al. 2018, 2019). In effect, depolarization continues until ionic equilibrium is reached across the membrane, leaving elevated extracellular K^+^ that prevents further recovery. While this disruption of signaling does not immediately lead to injury, prolonged or excessive cooling disrupts physiological processes, and if not mitigated, ultimately leads to cell death and, in turn, organismal death (Bayley et al. 2018).

### Cognitive mechanisms: retrograde amnesia

Because cooling has long been used as a tool to probe memory processes, the term “amnesia” provides a useful framework. Before spreading to other fields, the term “amnesia” was first used in the late 1800 s by clinical psychologists to describe a loss of or inability to retain information following an “event” (Lafleche and Verfaellie 2011). Many different factors and disruptions in biological function can cause amnesia in this broad sense, including both physical damage and a simple disruption of neural activity (Lafleche and Verfaellie 2011). Studies raising concerns about retrograde amnesia in bees were initially designed to disrupt memory consolidation using cooling, to assess short-term memory (Erber 1976; Erber et al. 1980). In one experiment, Erber (1976) gave bees the opportunity to explore blue and green illuminated feeding discs, only one of which contained sucrose. Bees were then marked and subjected to cooling, CO_2_, N_2_, or electroconvulsive shock before being released. Upon returning to the feeding station, bees from treated groups approached both discs equally, whereas untreated controls strongly preferred the color previously paired with sucrose. This effect occurred only if the treatment was administered within 7 min of the initial exploration. If administered after 7 min, groups performed like controls.

Erber et al. (1980) extended this work using a classical conditioning assay on restrained bees. Odors were paired with sucrose rewards, and a cooling probe was applied to specific brain regions. Cooling the mushroom bodies and olfactory lobes both disrupted anticipatory proboscis extension to the same extent as whole-body cooling, suggesting that general cooling transiently disrupts activity of these brain regions. As in the earlier study, effects were seen only if cooling occurred within 7 min, consistent with disruption of memory consolidation rather than tissue damage.

Together, these experiments show that cooling can transiently disrupt memory consolidation without causing lasting tissue damage. With this mechanistic context in mind, we next consider how cooling anesthesia has been applied in honey bee research, beginning with its earliest use in the 1980s.

## Studies of Cooling Anesthesia in Honey Bees

Building on these mechanistic insights, researchers have applied cooling anesthesia in a variety of experimental contexts. Here we review the earliest applications in the 1980 s and trace how later studies have evaluated its effects and safety.

### Early applications (1980s)

Cooling has been used because it was assumed to be safer than CO_2_, which was found to have harmful impacts. However, early studies indicated that cooling was not without potential risks. In the 1980 s, bees were typically cooled at −20 °C for ~ 3 min. The results were mixed and did not provide clear evidence of safety. The study most favorable to cooling methods was by Ebadi et al. (1980), who compared cooling bees at −20 °C for 3 min using specialized dry ice chambers in the field with several CO_2_ treatments. Bees were caught, immobilized, marked, and released, after which homing ability, pollen foraging, and mortality were assessed for one week. No significant effects on homing ability, foraging behavior, or longevity were found.

Other investigations, however, reported effects of cooling anesthesia on behavior and survival. Mardan and Rinderer (1980), using a similar treatment (−20 °C for 3 min; type of cooling and container not reported), tested hoarding behaviors—moving sucrose from a vial to an empty section of comb in their cage. They found that cooling significantly decreased hoarding, indicating behavioral impairment. Robinson and Visscher (1984) then tested −20 °C for 2 min in a freezer on newly emerged bees with a resulting 85% mortality rate, showing that age strongly influences cold tolerance. Together, these early papers establish cooling as a possible alternative to CO_2_, but also highlighted behavioral costs, age-dependent mortality, and unresolved questions about its overall safety. Although cooling was occasionally used in the 1990 s, we found no systematic studies from that decade evaluating its effects.

### Contemporary studies (2000–present)

Research attention to cooling anesthesia resumed in the early 2000 s, with Pankiw and Page (2003) and Chen et al. (2008). Pankiw and Page (2003) found that handling method significantly affected sucrose responsiveness. Thirty minutes after handling, their statistical analysis suggested that bees anesthetized with CO_2_ or chilling (5 °C for 4 min in glass vials) were more responsive than unanesthetized controls, even though the control group’s curve at 30 min appeared left-shifted and thus more responsive at high sucrose concentrations. By 60 min, all groups had converged. The authors interpreted this pattern as anesthesia masking the transient handling stress evident in unanesthetized bees.

Chen et al. (2008) cooled bees for 4 min at 5 °C (noting immobility at 1 min) to test the effects of cooling on the levels of dopamine (involved in memory retrieval), octopamine (involved in memory consolidation), and serotonin (associated with modulatory and regulatory functions). Results showed that cooling significantly lowered dopamine and octopamine levels, while serotonin was unaffected. Because measurements were taken only immediately after cooling, the duration of these neuromodulator changes remains unclear.

Chen et al. (2014) further examined cooling effects on associative olfactory proboscis extension learning. Bees were cooled for 90 s on ice on tinfoil, resulting in ~ 3 min of immobility, and then trained to associate an odor and a sucrose reward. Cooling 30 min before training (acquisition) or immediately after training (consolidation) significantly impaired performance, whereas cooling during retrieval had no effect. This aligns with earlier work (Erber 1976), which showed chilling disrupts memory consolidation only if applied shortly after training. When Chen et al. (2014) allowed a 60-min recovery period before training, no significant differences were observed between cooled and untreated bees. Locomotor assays in the same study also showed reduced activity at 15 min after treatment, but full recovery by 30 min. These results suggest that recovery intervals of 30–60 min may be sufficient for many behavioral assays under the conditions tested.

Frost et al. (2011) attempted to test a variety of cooling methods on sucrose responsiveness. However, as they themselves noted, their experimental design confounded satiation with the time interval since cooling, making results difficult to interpret. Replication with improved controls would be valuable. Together, studies from the 2000 s show that cooling can affect responsiveness, neuromodulator levels, learning, and locomotion, and that recovery time is often critical. Whether apparent behavioral recovery truly reflects full physiological recovery, however, remains uncertain—an issue addressed in the next section.

### Potential harm in current methods

While information retention may not be a primary concern, questions remain regarding tissue damage and the reporting of methods. Tutun et al. (2020) recently examined the effects of various cooling treatments for various durations, including refrigeration (4 °C) and exposure to one of two freezers (−20 °C and −80 °C). Cooling on ice, another common method, was not tested. Bees were grouped together in cages in groups of 20, cooled for their assigned duration, and then scored as a collective based on their degree of anesthetization. Time to full recovery was recorded, and mortality was assessed after 2 h at room temperature. The authors concluded that 3 min at −20 °C was “safe” despite a 2.5% mortality rate.

Although this loss may seem minor, mortality alone does not provide a complete assessment of safety. Survivors may still experience tissue damage, as shown in locusts, where cooling produced neural and muscular injury even in individuals that appeared to recover behaviorally (MacMillan et al. 2015). Thus, mortality or overt behavioral recovery alone may not rule out the possibility of chill injury under these treatments. In addition, physiological measures themselves may be affected by cooling. Lin et al. (2004) showed that cold anesthesia and related handling stress can alter juvenile hormone titers in worker honey bees. Impacts of cooling on titers was found to vary greatly between colonies. Nurse-age bee titers rose significantly after as little as 1 h of cooling in some colonies. Forager-age titers, however, rose significantly after 8 h of cooling (in 1 colony), significantly fell after 24 h of cooling (in 2 colonies), or remained constant after 24 h of cooling (in 2 colonies). Being mindful of potential changes in juvenile hormone titers as a result of cooling or handling is particularly important because juvenile hormone is widely used as a physiological marker in studies of behavioral maturation and division of labor, and chilling has often been used as a routine immobilization method in this literature (e.g., Sullivan et al. 2000, 2003).

## Current Reporting Practices in Cooling Studies

Few papers have directly examined how consistently cooling methods are reported, beyond the key experimental studies already reviewed (e.g., Pankiw and Page 2003; Lin et al. 2004; Chen et al. 2008, 2014; Frost et al. 2011; Tutun et al. 2020). Frost et al. (2011) provided an excellent overview more than a decade ago, but the state of reporting appears to have changed little since. Variations in temperature, duration, container material, and cooling method (freezer, refrigerator, ice, etc.) could all influence outcomes, yet such details are often missing.

**Figure 1.**
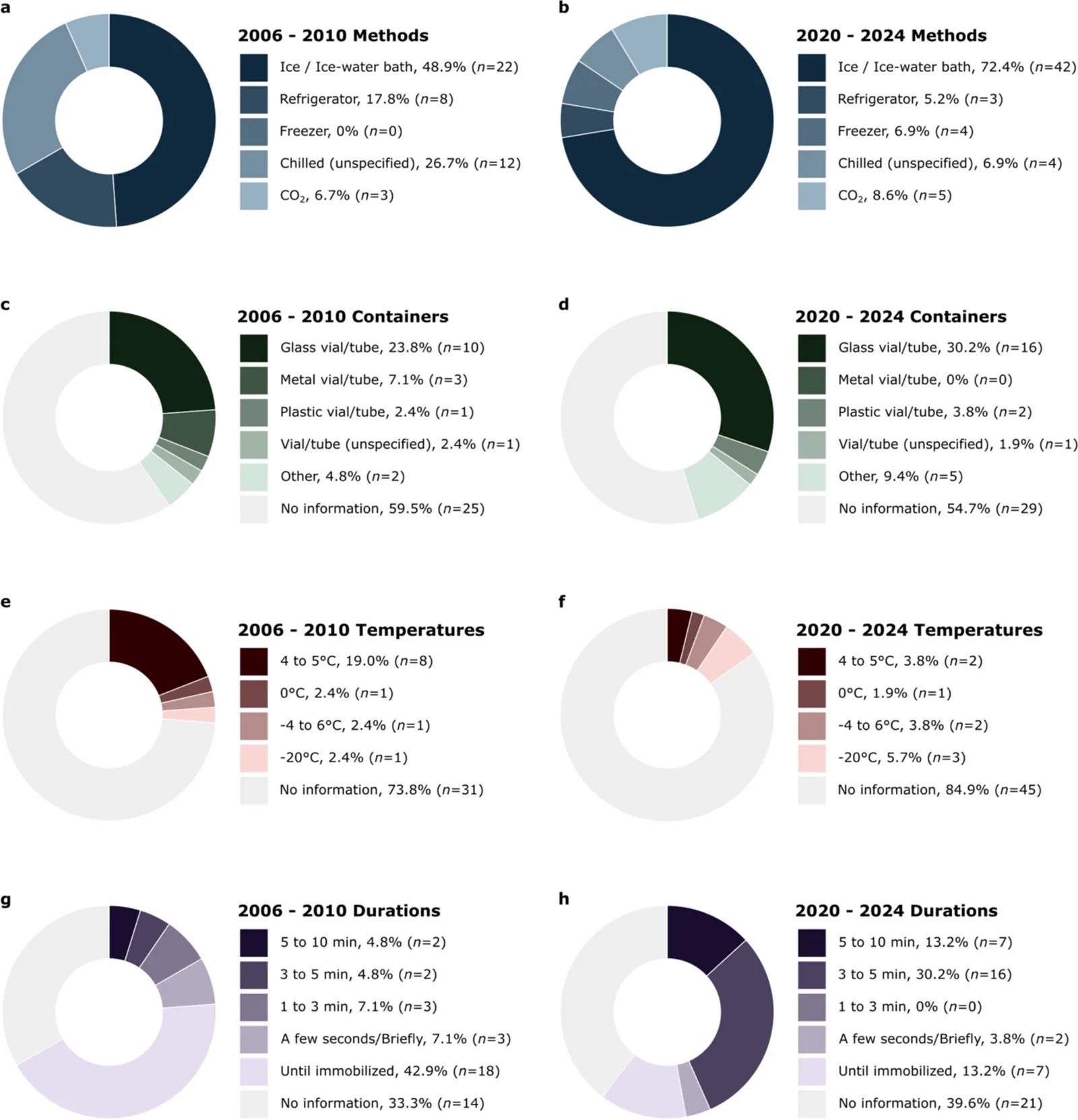
Percentages of anesthetization methods used on honey bees in primary research articles between 2006–2010 (a, c, e, g) and 2020–2024 (b, d, f, h). Counts for panel (a) (n = 53) and (b) (n = 58) include all methods listed in the relevant literature found. Panels (c) to (h) include exclusively papers that reference cooling anesthetization (2006–2010: n = 49; 2020–2024: n = 53). Search terms are provided in Appendix Table A.1.

To assess the extent of this problem, we searched the Scopus database (http://www.scopus.com) for honey bee harnessing and anesthetization studies across two five-year periods, 2006–2010 and 2020–2024, bracketing Frost et al.’s (2011) call for improved transparency (Table A.1). Cooling was overwhelmingly the method of choice in both eras, but reporting of how it was applied was inconsistent. Many studies failed to specify container type, temperature, or duration, and general phrasing such as “chilled on ice” was frequently used (Fig. 1). Overall, methodological transparency has shown little improvement over the past decade.

### Inconsistencies in reporting

When details were provided, they were inconsistent across the four main parameters (Fig. 1). Methods most often involved ice or ice-water baths, but many papers gave no further information and simply stated that bees were “chilled” (Fig. 1a–b). Phrases such as “immobilized on ice” were common, but ambiguous: in some cases, this may have referred to submersion in an ice bath, in others to bees placed on ice. Without clarification, these methods are not readily comparable; temperatures and thus the intensity of treatment can vary. The type of container holding the bee during chilling procedures was also frequently omitted, with glass occasionally specified, though plastic, metal, and other materials were also reported (Fig. 1c–d). Container material can be an important detail: metals conduct heat much faster than plastic or glass, and the size of the container alters how quickly internal air equilibrates to the cooling source. Both factors can change how rapidly a bee’s body temperature drops, yet these variables were seldom documented.

Temperature was among the least consistently reported details: in most cases, no value was given, and where it was, the range spanned from −20 °C to 5 °C (Fig. 1e–f). Finally, durations varied widely, from a few seconds to 10 min, but were often described only as “until immobilized,” a vague endpoint that makes comparisons across studies difficult (Fig. 1g–h).

Further, since many methods (e.g. ice baths, refrigerators, freezers) require bees to be removed from treatment to check their state, the exact point of chill coma onset often cannot be verified. Such imprecision makes it difficult to know how long individuals had already been immobilized before removal. Overall, the most reliable reporting occurred when authors specified a defined range of cooling times and described the materials used to contain bees. However, these details were absent in the majority of studies, limiting replication and comparability. These inconsistencies provide important context for assessing whether reporting has improved over time.

### Comparisons between time periods

Comparing the two survey windows reveals modest improvements in reporting but continued shortcomings (Fig. 1). Cooling was the predominant anesthetic method in both periods, but its documentation shifted in some important ways. The proportion of studies giving no details beyond “chilled” dropped from 26.7% in 2006–2010 to 6.9% in 2020–2024, while explicit references to ice or ice-water baths increased correspondingly, 48.9% to 72.4% (Fig. 1a–b). Duration reporting also changed noticeably. “Until immobilized” was the most common description in the earlier period (42.9%) but fell sharply in recent years (13.2%). In its place, fixed intervals of 3–5 min became more common, rising from just 4.8% to 30.2% of studies (Fig. 1g–h). This shift suggests a growing awareness of the need for more consistent descriptions, even if exact replication remains elusive.

In contrast to the modest improvements in method and duration reporting, container and temperature details showed little change over time (Fig. 1c–f). More than half of studies in both periods failed to specify container material, and when mentioned, glass was usually the default with only scattered reports of plastic, metal, or other materials. Temperature reporting was even less common, and the few values provided spanned from −20 °C to 5 °C. Overall, these categories remain among the least consistently documented parameters examined, with no clear progress between decades.

## Conclusions

Overall, while cooling is an important tool within honey bee research and indeed in many invertebrate studies, relatively little comparative work has systematically evaluated the range of cooling methods currently employed in neuroethological research. Existing studies show that cooling can influence hoarding behaviors, neuromodulator levels (including octopamine and dopamine), juvenile hormone titers, learning acquisition and memory consolidation, and locomotion (Erber 1976; Erber et al. 1980; Mardan and Rinderer 1980; Lin et al. (2004); Chen et al. 2008; Chen et al. 2014). While several of these experiments reported apparent recovery within 1 h following cooling, none systematically examined the full range of methods now common in the literature. Reporting practices appear to have improved modestly in recent years, but important gaps remain that complicate reproducibility and interpretation. Clearer reporting would facilitate comparison across studies and help determine whether observed effects reflect experimental manipulation or anesthetic handling itself. Without consistent reporting of cooling conditions, it remains difficult to assess whether differences across studies reflect biological phenomena or methodological variation.

Alternative anesthetics have been investigated, such as isoflurane and sevoflurane in *Drosophila* (MacMillan et al. 2017). In honey bees, however, the use of chemical anesthetics has largely been limited to studies evaluating anesthetic effects rather than their adoption as tools in neuroethological experiments. Isoflurane has been shown to influence circadian rhythm (Cheeseman et al. 2012; Ludin et al. 2016) and to have a reduced metabolic impact relative to cooling and CO_2_ (Gooley and Gooley 2023). However, short- and long-term effects on behavior, lifespan, and neuromodulators remain incompletely characterized. Sevoflurane remains to be tested in honey bees.

Future work directly comparing the cooling methods reported in the literature, across temperatures, durations, and container types, would improve interpretation of both past and future studies. Measuring impacts on neuromodulators, locomotor activity, learning and memory, and tissue integrity across multiple time points and temperatures would clarify the dynamics of recovery following cooling. Understanding these biological effects remains essential for interpreting existing results, even if cooling is phased out as an anesthetic method. Anesthetic effects should also be considered alongside handling stress itself, which in some contexts has been shown to exceed the effects of CO_2_ or cooling on sucrose responsiveness (Pankiw and Page 2003). Likewise, simply caging bees without cooling prior to drawing hemolymph was found to have similar or greater effects on juvenile hormone titers as cooling (Lin et al 2004). Thus, anesthesia remains a useful tool when appropriately applied. The challenge lies in understanding and mitigating its effects so researchers can select methods that minimize disruption for their experimental goals. More broadly, recognizing cooling itself as an experimental factor, rather than a neutral preparatory step, is essential for ensuring reliable and physiologically meaningful outcomes in future honey bee research.

In light of these considerations, several practical steps may improve the reliability and comparability of future studies. At minimum, researchers should clearly report cooling conditions, including temperature, duration, method of cooling (e.g., ice, refrigeration, or freezer exposure), and containment type. Cooling protocols should be selected and applied consistently across individuals and trials, and recovery periods standardized within studies to reduce variation attributable to handling. Across the literature, commonly used approaches include short-term cooling on ice and recovery periods prior to behavioral testing, although the effects of these practices remain incompletely characterized. Where feasible, pilot validation of cooling protocols for specific experimental outcomes (e.g., neuromodulator levels, learning performance) would help determine whether observed effects reflect the experimental treatment or anesthetic handling. When such validation is not possible, these potential effects should be acknowledged as a source of variability when interpreting results.

## Data Availability

No original data were generated or analyzed for this review article. The list of articles included in the literature survey can be reproduced using the Scopus search query described in Table A.1.

## Appendix A

**Table A.1:** Scopus search terms for the five whole years prior to Frost et al. (2011) and the most recent whole five years (2006–2010 and 2020–2024, respectively)

Time period	Search string
2006–2010	(TITLE ("apis mellifera" OR "honey bee" OR "honeybee") AND TITLE- ABS-KEY ("anesthesia" OR "anesthesia" OR "anesthetic" OR "anaesthetic" OR "anesthetize" OR "anesthetize" OR "proboscis extension response" OR "proboscis extension reflex" OR "harness" OR "harnessing")) AND PUBYEAR > 2005 AND PUBYEAR < 2011 AND (LIMIT-TO (DOCTYPE, "ar"))
2020–2024	(TITLE ("apis mellifera" OR "honey bee" OR "honeybee") AND TITLE- ABS-KEY ("anesthesia" OR "anesthesia" OR "anesthetic" OR "anaesthetic" OR "anesthetize" OR "anesthetize" OR "proboscis extension response" OR "proboscis extension reflex" OR "harness" OR "harnessing")) AND PUBYEAR > 2019 AND PUBYEAR < 2025 AND (LIMIT-TO (DOCTYPE, "ar"))

Counts in Fig. 1 reflect a curated dataset; studies where anesthetization methods themselves were the primary experimental focus, or where search terms yielded irrelevant results, were excluded
